# Long Non-Coding RNA Landscape in Prostate Cancer Molecular Subtypes: A Feature Selection Approach

**DOI:** 10.3390/ijms22042227

**Published:** 2021-02-23

**Authors:** Simona De Summa, Antonio Palazzo, Mariapia Caputo, Rosa Maria Iacobazzi, Brunella Pilato, Letizia Porcelli, Stefania Tommasi, Angelo Virgilio Paradiso, Amalia Azzariti

**Affiliations:** 1 Molecular Diagnostics and Pharmacogenetics Unit, IRCCS IstitutoTumori Giovanni Paolo II, 70124 Bari, Italy; m.caputo@oncologico.bari.it (M.C.); b.pilato@oncologico.bari.it (B.P.); s.tommasi@oncologico.bari.it (S.T.); 2 Laboratory of Nanotechnology, IRCCS IstitutoTumori Giovanni Paolo II, 70124 Bari, Italy; a.palazzo@oncologico.bari.it; 3 Laboratory of Experimental Pharmacology, IRCCS Istituto Tumori Giovanni Paolo II, 70124 Bari, Italy; r.m.iacobazzi@oncologico.bari.it (R.M.I.); l.porcelli@oncologico.bari.it (L.P.); a.azzariti@oncologico.bari.it (A.A.); 4 Scientific Directorate, IRCCS Istituto Tumori Giovanni Paolo II, 70124 Bari, Italy; a.paradiso@oncologico.bari.it

**Keywords:** prostate cancer, lncRNA, feature selection

## Abstract

Prostate cancer is one of the most common malignancies in men. It is characterized by a high molecular genomic heterogeneity and, thus, molecular subtypes, that, to date, have not been used in clinical practice. In the present paper, we aimed to better stratify prostate cancer patients through the selection of robust long non-coding RNAs. To fulfill the purpose of the study, a bioinformatic approach focused on feature selection applied to a TCGA dataset was used. In such a way, LINC00668 and long non-coding(lnc)-SAYSD1-1, able to discriminate ERG/not-ERG subtypes, were demonstrated to be positive prognostic biomarkers in ERG-positive patients. Furthermore, we performed a comparison between mutated prostate cancer, identified as “classified”, and a group of patients with no peculiar genomic alteration, named “not-classified”. Moreover, LINC00920 lncRNA overexpression has been linked to a better outcome of the hormone regimen. Through the feature selection approach, it was found that the overexpression of lnc-ZMAT3-3 is related to low-grade patients, and three lncRNAs: lnc-SNX10-87, lnc-AP1S2-2, and ADPGK-AS1 showed, through a co-expression analysis, significant correlation values with potentially druggable pathways. In conclusion, the data mining of publicly available data and robust bioinformatic analyses are able to explore the unknown biology of malignancies.

## 1. Introduction

Prostate cancer (PCa) is one of the most common cancers in men. It is the second-leading cause of death for men in the US [1], and although, for 2020 compared to the last five years, its incidence has decreased, it remains the third-leading cause of death in Europe [2]. Advanced age, ethnicity, genetic factors and family history are well-established risk factors for PCa [3,4], while diet, obesity, physical inactivity, hyperglycemia and environmental pollution are positively associated with prostate cancer [5,6]. Usually, monitoring of the plasmatic prostate-specific antigen (PSA) level in the blood is used for the check-up of the health state of the prostate, and if the value is higher than 4 ng/mL, it is a possible risk factor for prostate cancer [7]. Since high PSA values do not necessary correspond to prostate cancer, a gland biopsy is a mandatory step. The correct staging of prostate cancer represents the starting point to establish which is the best therapeutic strategy, to obtain information about the prognosis and to compare the results of the various therapeutic options. Multiple clinical and biopsy parameters can contribute to staging, especially if added within specific nomograms or through predictive models [8]. 

The standard treatment of prostate cancer has different objectives, depending on the anatomical extension and aggressiveness of the disease but, also, on the patient’s life expectancy and the presence of comorbidities that may represent a risk of death higher than that represented by prostate cancer. For this reason, a “watchful waiting” policy (surveillance in the absence of systematic checks) may be indicated in patients with a short life expectancy (generally, less than 10 years). Similarly, patients suffering from a very low low-risk disease, even in the presence of a good life expectancy, can be directed towards an “active surveillance policy”. In patients with metastatic disease, palliation remains the most concretely achievable goal, especially if symptomatic. For these patients, there are currently various hormone therapy (LH-RHa (luteinizing hormone-releasing hormone agonist) ± nonsteroidal antiandrogen ± Docetaxel, LH-RHa + Abiraterone and LH-RHa antagonist) [9,10,11,12,13,14] and chemotherapy (Docetaxel) [15,16,17] options that, together with the most recent forms of radiometabolic therapy (alpha emitters) and bone-targeted therapies, can significantly impact both their quality and their life expectancy. Prostate cancer has long-been considered a low chemo-sensitive tumor, but in the early 2000s, some controlled studies demonstrated the effectiveness of Docetaxel in patients suffering from castration-resistant disease (CRPC) [18]. For patients affected by CRPC who progress after first-line treatment with deprivation androgenic (ADT), the treatment options available have increased considerably within the past few years and include new chemotherapeutics, in addition to Docetaxel [19,20], new hormonal therapies [21], radio-compounds (Radium-223) [22,23] and immunological therapies (Sipuleucel-T, a dendritic cell vaccine) [24]. However, immunotherapy, with checkpoint inhibitors such as ipilimumab and nivolumab [25] has so far produced disappointing results in the treatment of prostate cancer, while the phase II study KEYNOTE-199, the results of which were presented at ASCO 2018, would seem to highlight a good therapeutic activity of pembrolizumab monotherapy [26].

In order to identify a personalized therapy based on the specific characteristics of patients with prostate cancer, an important issue to consider is that PCa shows a high grade of genomic variegation, with different patterns and clinical implications. Usually, the genomic alteration occurs in the early stage of the tumor and accumulates as it progresses; therefore, is possible to distinguish different molecular subtypes of PCa on the basis of gene fusion, gene expression signature and other molecular alteration, but this classification does not correspond to an accurate and precise tumor staging or predictive/prognostic information [27]. TMPRSS2-ERG fusion is the most common molecular alteration in localized PCa, with a frequency of 40–50% of all prostate cancer diagnosed [28]. However, other gene fusions are grouped in the ETS-positive subclass. This includes fusions with ETS transcription family genes like ETV1 (10%), ETV4, ETV5 and FLI1 (1–5%). ETS-negative prostate cancer shows recurrent mutations in the SPOP, FOXA1 and IDH genes. Given the high molecular heterogeneity of prostate cancer, a series of both ETS-positive/negative subclasses are generally classified as “others”. In this macro-category, the less recurrent, usually with an unknown molecular meaning, are grouped [29]. 

The integrated use of an advanced genome analysis has allowed to recognize and to identify ncRNA, which, even if they do not encode for proteins, have specific biological functions in cancerogenesis and metastasis. Among ncRNAs, the aberrant expression of some long non-coding RNA (lncRNAs) are also correlated with the disease state for PCa [30,31] and with a possible role in competing endogenous RNAs (ceRNAs) [32], suggesting them a role as targets for therapeutic intervention. Long non-coding RNAs are RNAs transcripts >200 nucleotides in length [33], with a role in cellular differentiation [34] and in cancer pathway [35] due to the influence of specific gene expression targets and could undergo a post-transcriptional processing to produce numerous 5′-cappelled small RNA [36]. 

The therapeutic approaches against lncRNAs could be direct by affecting lncRNA expression or indirect by targeting protein-coding genes dysregulated by the lncRNA with a consequent perturbation of its molecular pathway and lethal effects on cancer cells [37]. Briefly, the strategies to target lncRNA encompass the repression of lncRNA transcription by utilizing DNA-binding elements that target its genomic locus, the silencing of the lncRNA to induce transcript degradation, the utilization of small molecules thatmask the binding site for lncRNAs, thereby disrupting the network of interactions responsible for the altered function in disease-related lncRNA, and the utilization of aptamers, which antagonize the lncRNA association with binding partners by folding into a three-dimensional structure with higher affinity and specificity for the same regions [38]. However, in our opinion, the not well-understood plethora of proteins and, consequently, of cellular pathways that each lncRNA could affect, considering also their ubiquitous presence, exclude the possibility to directly target lncRNAs for the biological effects not easy predictable and perhaps potentially dangerous for noncancer cells. 

In the present paper, a feature selection approach was applied to a TCGA-PRAD cohort to identify lncRNA able to stratify ERG-positive cases and other subtypes. Moreover, the same approach was also used to focus on the cases with no peculiar genomic alterations.

## 2. Results

### 2.1. lncRNA Discriminating ERG-Positive Subtype: Feature Selection

The lncRNA expression data (*n* = 13,074) were obtained from HT-Seq counts data of the TCGA-PRAD cohort on the basis of LNCPipedia v5.2 annotation. The TCGA consortium described the “molecular taxonomy” of prostate cancer (PCa), suggesting eight molecular subtypes. In Table 1, it can be observed that ERG-positive patients include almost 50% of the entire cohort. 

**Figure 1:**
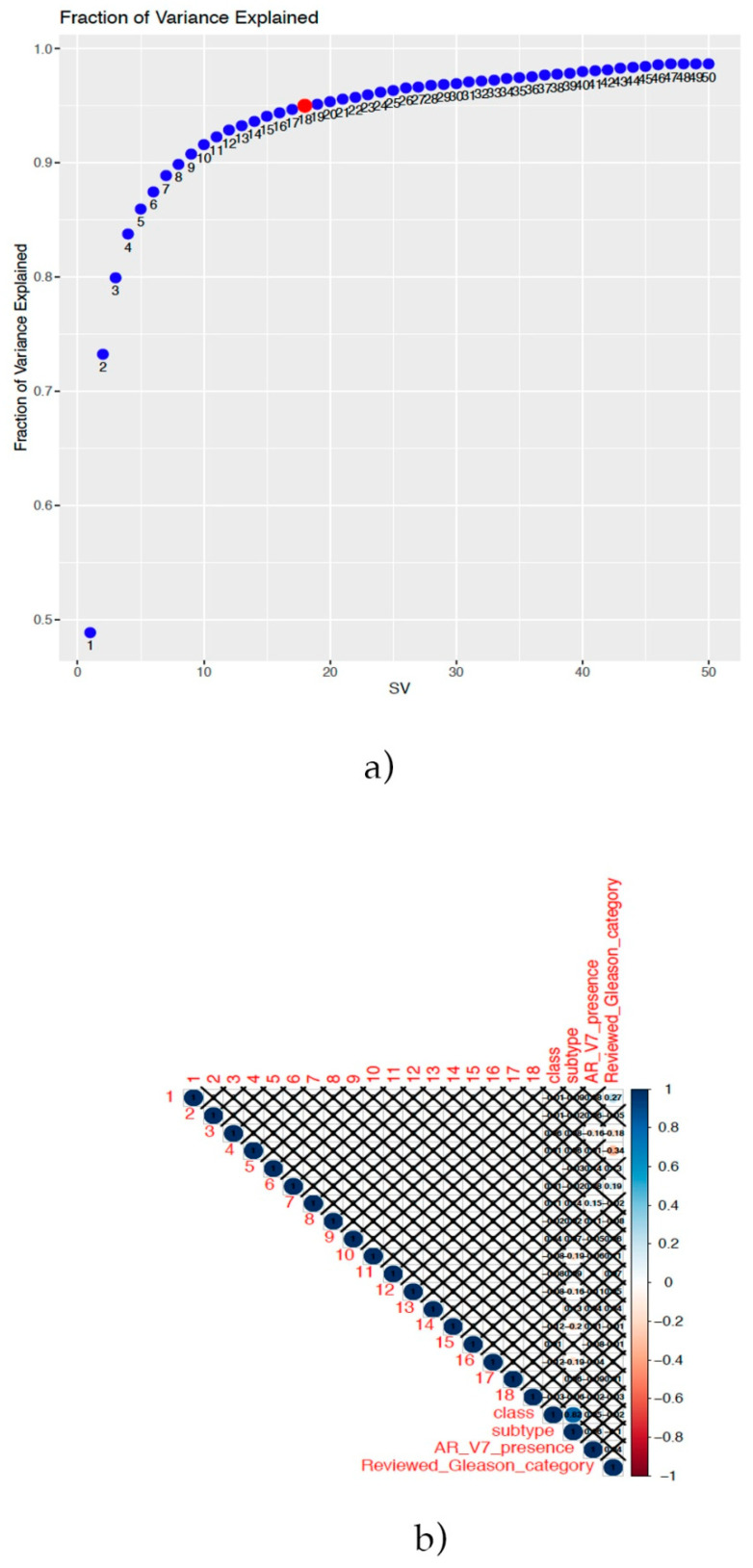
(**a**) Variance explained by the surrogate variables (SVs) identified. The red dot indicates that 18 SVs explain 95% of the variance. (**b**) Correlation plot between the SVs and known variables. It is important to note than none of them were significantly correlated with the class variable (ERG/not-ERG).

Thus, aiming to identify lncRNAs able to discriminate ERG-positive tumors from the other subtypes, the DaMiRseq pipeline was applied. Firstly, after lncRNA expression data filtering by read counts and the coefficient of variation (see “Materials and Methods”), 10,810 features were filtered out, including 25 hyper-variants. After, sample filtering, the sample size was 329 cases. The SVA algorithm indicated that 18 surrogate lncRNAs were able to explain 95% of the variance (Figure 1a), and the correlation plot suggested that none of them had to be excluded in order to adjust the data, because no correlation with the “class” variable was detected, which was the important result to be considered. The correlations with other variables were not considered in order to avoid overcorrection of the data (Figure 1b).

**Figure 2:**
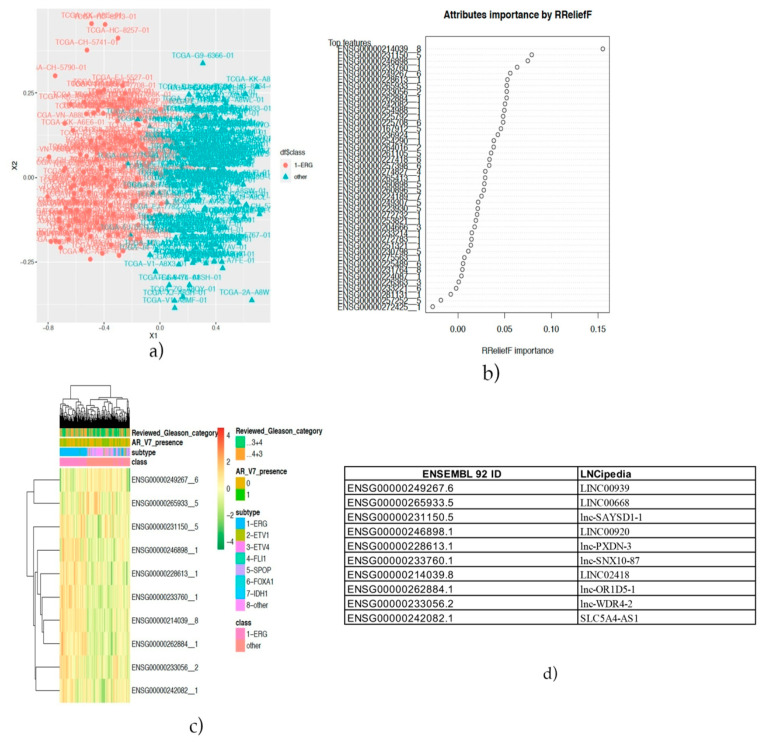
(**a**) Multidimensional scaling plot that shows the effects of normalization and data adjustment. (**b**) Feature importance plot showing the best performing long non-coding RNAs (lncRNAs). (**c**) Cluster gram of the best top 10 lncRNAs discriminating ERG/not-ERG subtypes, and (**d**) an Ensembl ID/LNCPipedia ID conversion table.

Finally, the supervised machine learning approach, described by Chiesa et al. [39], was used to accomplish our aim, which was the selection of robust features—in this case, lncRNAs to stratify ERG-positive cases from negatives ones. The MDS (Multi Dimensional Scaling) plot in Figure 2a shows sample distances after feature selection and filtering of highly correlated lncRNAs. The selected features were then ranked by importance (Figure 2b), and the best 10 predictors that were identified were depicted in a cluster gram (Figure 2c). It could be observed that ERG-positive and not are located in two different clusters. Notably, no pattern of separation for ARv7 presence/absence and Gleason scores was evidenced.

**Figure 3:**
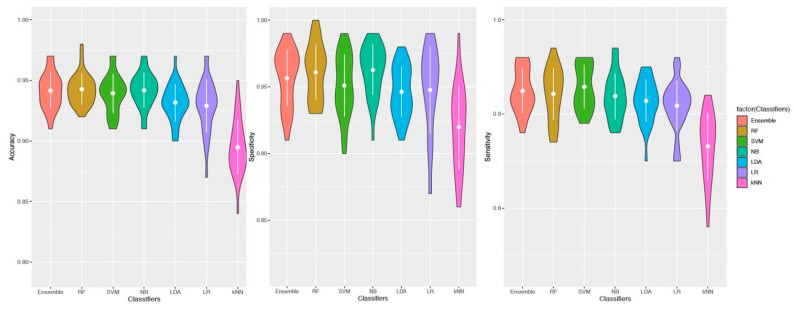
Violin plot depicting the accuracy, specificity and specificity of the classification algorithms.

The classification performances of the best 10 predictors were estimated using a meta-learner—namely, “Ensembl”—that combines the RF, SVM, NB, LDA, LR, and kNN classifiers. Elevated accuracy, sensitivity and specificity were always greater than 90% for all classifiers but the kNN algorithm (Figure 3).

### 2.2. lncRNA-mRNA Coexpression Analysis of lncRNA Discriminating ERG-Positive Subtype

The biological role of the best 10 lncRNAs was explored through the creation of a co-expression matrix. The lncRNAs and mRNAs were significantly correlated (r > |0.6| and *p*-value < 0.05) extracted. In Figure 2d, a conversion table of 92 Ensembl IDs and gene symbols are reported. 

In Table 2, co-expressed lncRNAs and mRNAs are reported. Thus, significantly correlated genes to lncRNA-linked mRNAs were also extrapolated to build up a network.

**Figure 4:**
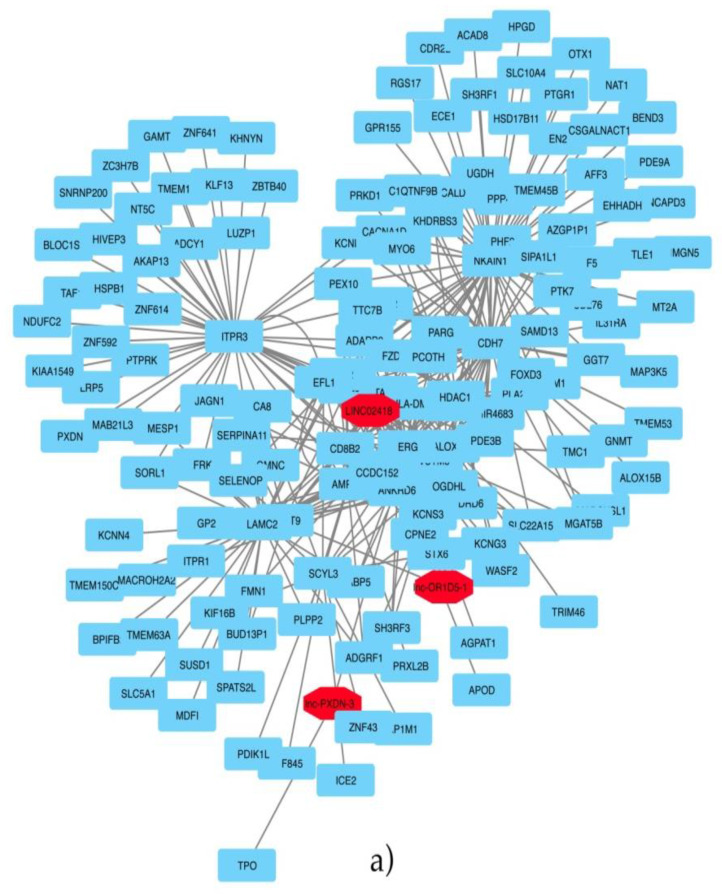

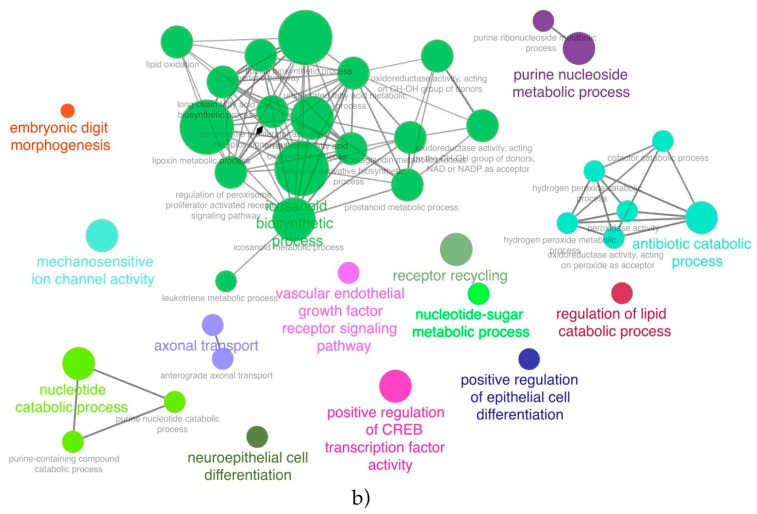
(**a**) Co-expression network, including significant correlation oflncRNAs/coding genes. Red cells indicate lncRNAs showing significant coexpression with mRNAs (**b**). Functional enrichment of the co-expression network.

In Figure 4a, it could be observed that LINC02418, lnc-OR1D5-1 and lnc-PXDN-3 showed significant correlations, and the relative biological enrichment network, depicted in Figure 4b, showed that VEGFR signaling pathways, CREB activity, purine metabolism and the regulation of differentiation of epithelial cells are enriched pathways. 

### 2.3. PPI Network and Identification of Highly Connected Subnetwork in ERG-Positive Subtype

**Figure 5:**
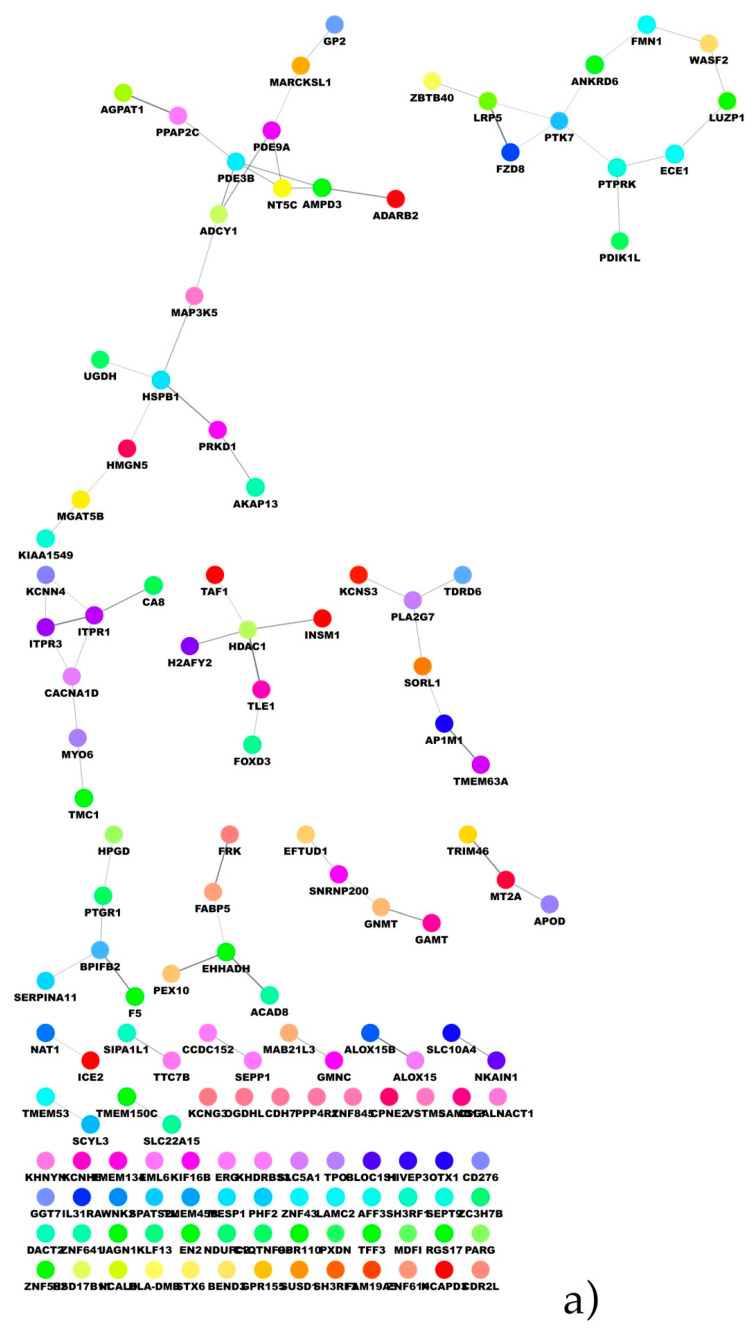

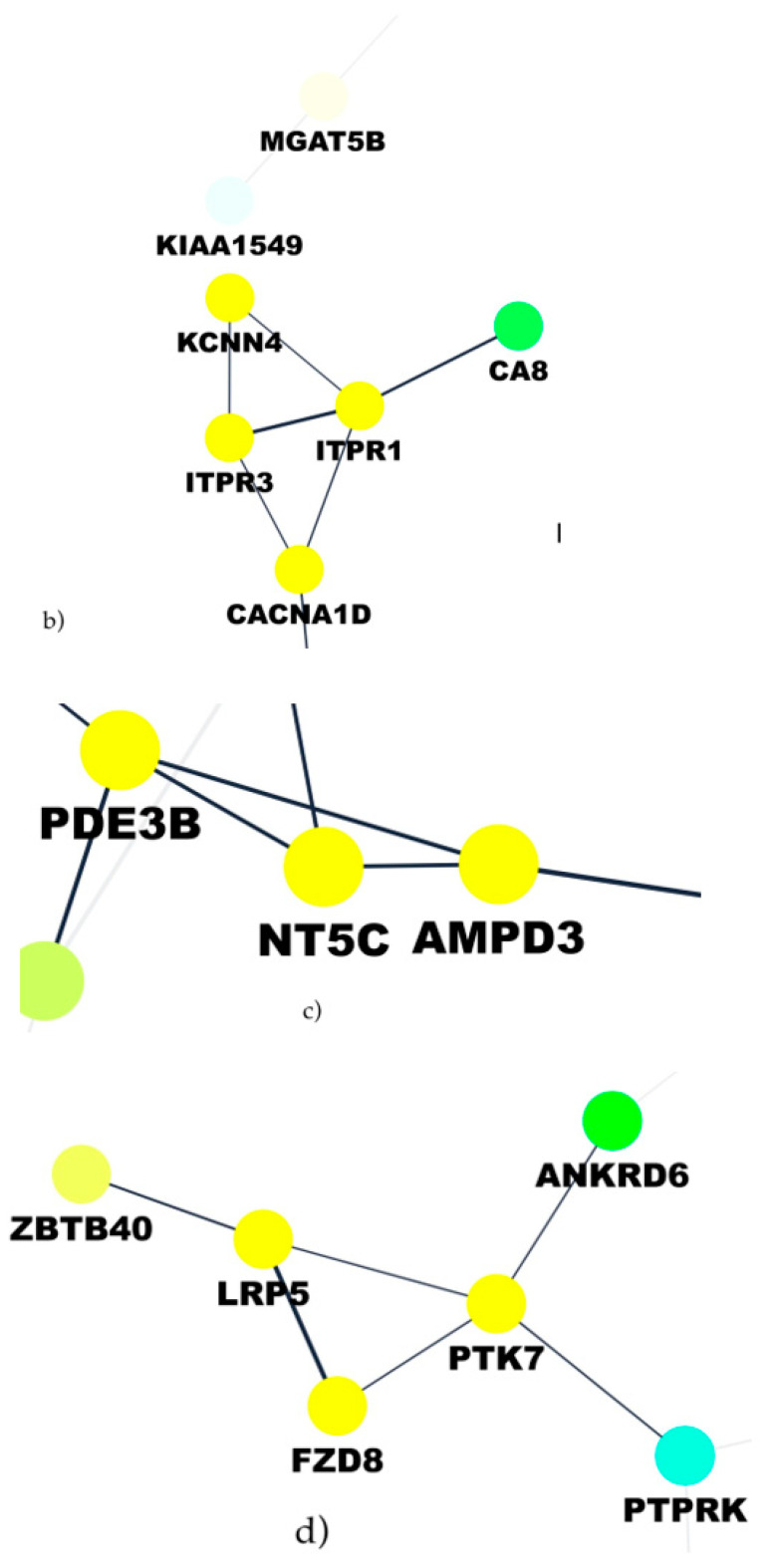
(**a**) Protein–protein interaction (PPI) network derived from the co-expression network. (**b**–**d**) Subnetworks identified by the Molecular Complex Detection (MCODE) algorithm.

A protein–protein interaction (PPI) network was built up through the STRING database and Cytoscape retrieval engine (Figure 5a). The network was then analyzed through the Molecular Complex Detection (MCODE) algorithm and identified three highly connected subnetworks. The first cluster included the KCNN4, ITPR3, ITPR1 and CACNA1D proteins (Figure 5b), enriched in pathways such as the GnRH (gonadotropin-releasing hormone) signaling pathway, calcium regulation and aldosterone synthesis (Table 3). 

The subnetwork including the PDE3B, NT5C and AMPD3 proteins (Figure 5c) is enriched in pathways mainly involved in purine metabolism (Table 4). The third PPI network cluster includes the LRP5, PTK7 and FZD8 proteins (Figure 5d), whose enrichment analysis indicated the Wnt pathway as the most enriched (Table 5).

### 2.4. Prognostic and/or Predictive Role of the Best 10 lncRNAs Discriminating ERG versus Non-ERG Subtypes

Clinical data of the TCGA-PRAD cohort was downloaded—in particular, the Gleason score and PFS time related to first-line treatment (hormone-therapy)—in order to hypothesize a possible role of the selected lncRNAs as prognosticators and/or predictors of the response to hormone therapy. 

**Figure 6:**
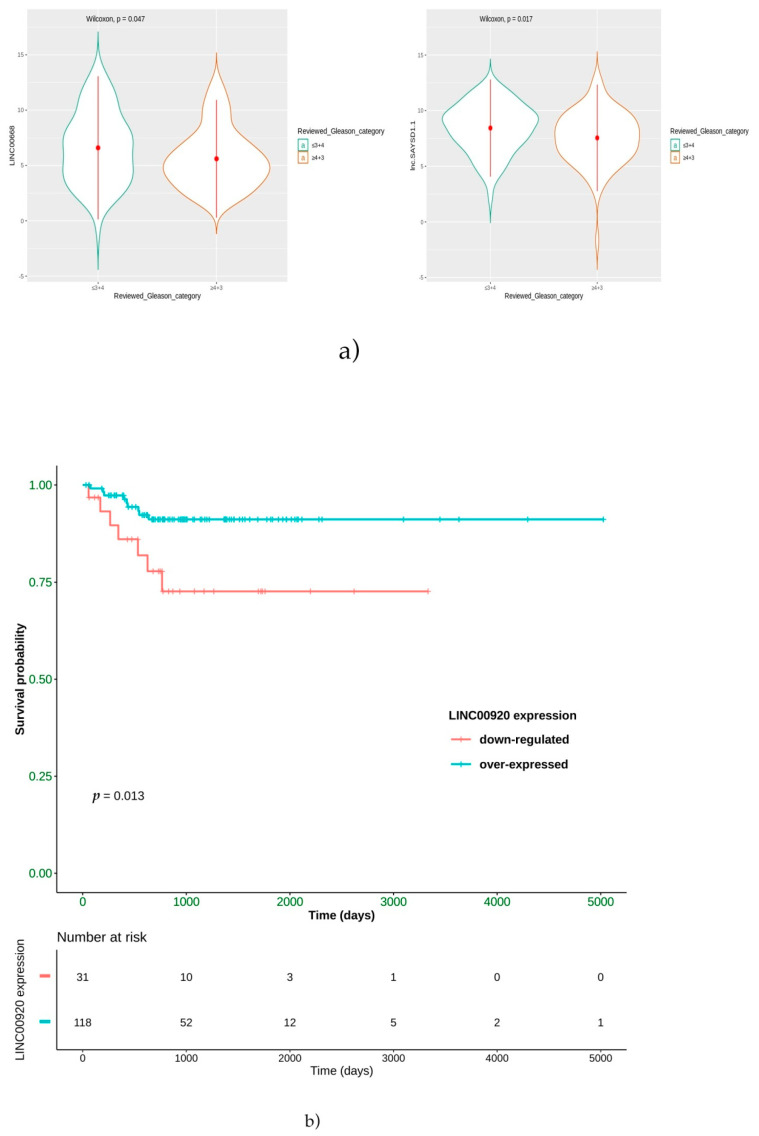
(**a**) Violin plots showing that ERG-positive patients with Gleason scores ≤ 3+4 overexpress LINC00668 and lnc-SAYSD1-1. (**b**) PFS Kaplan-Meier curve stratifying the ERG subset according to the LNC00920 expression.

The prognostic role was explored by stratifying patients according to their Gleason scores in both the ERG-positive and non-ERG subgroups. In particular, as demonstrated by the literature, patients with Gleason scores ≤ 3+4 are low-grade, whilst PCa cases with Gleason scores ≥ 4+3 are affected by high-grade tumors and, thus, with a worse prognosis. In our analysis, as shown in Figure 6a, only patients with a Gleason score ≤ 3+4 significantly overexpressed LINC00668 and lnc-SAYSD1-1 in the ERG-positive subgroup. In contrast, no positive associations were found in the non-ERG one. Thus, it could be supposed that these two lncRNAs could have a positive prognostic role inERG-positive PCa patients.

Furthermore, patients were stratified according to the expression values of the best 10 lncRNAs and by log-rank test Kaplan-Meier curves. Importantly, we demonstrated that a low level of only LINC00920 lncRNA was strongly correlated with a poorer PFS after hormone therapy (*p* < 0.013) in the ERG-positive subgroup of PCa patients; conversely, this correlation was absent in the non-ERG subgroup (Figure 6b).

### 2.5. Feature Selection to Classify the “Not-Classified” Subtype

**Figure 7:**
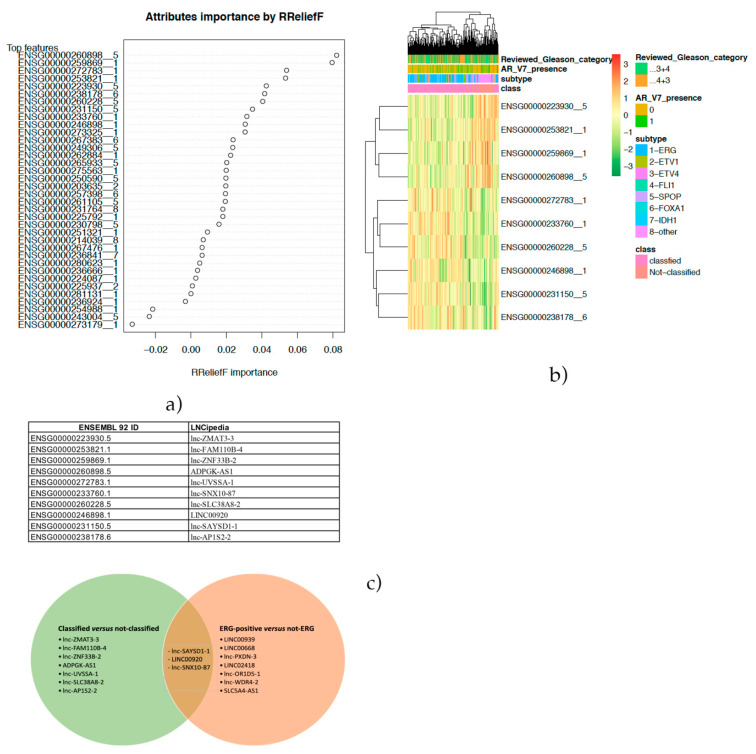
(**a**) Dot plot representing lncRNAs ranked by their importance. (**b**) Cluster gram of the best top 10 lncRNAs discriminated by classified/not-classified prostate cancer (PCa) cases. (**c**) Ensembl ID/LNCipedia ID conversion table and Venn diagram including top 10 ERG/not-ERG lncRNAs and classified/not-classified lncRNAs.

The authors of the PCa molecular subtyping identified the so-called “others” subgroup [27], constituting almost 25.9% of the entire cohort (Table 1). Such a group, that we define as the “not-classified” group, showed no peculiar genomic alterations. Thus, to be able to classify this group, the same pipeline was applied, using as the class variable the “classified” group, including ERG, ETV1, ETV4, FLI1, SPOP, FOXA1 and IDH1-positive patients, and “not classified”, including, as stated above, the “others” subtype. The lncRNA raw read count expression matrix was filtered for lncRNA expression. In particular, in this step, 10,773 features were filtered out, including 32 hyper-variant features. Thus, 2267 features remained and underwent a variance stabilizing transformation (“vst”) normalization. In the sample filtering step, three samples were discarded, and 328 patients remained. Twenty surrogate variables were identified as able to explain 95% of the variance and, thus, were used to adjust the data. In the feature selection step, 2229 lncRNAs were discarded, leaving 38 features. Moreover, two highly correlated lncRNAs were removed, and thus, 36 features remained for the classification. The remaining features were ranked for their importance, as shown in Figure 7a, and the top 10 best lncRNAs were selected. The clusterplot (Figure 7b) evidenced a clear clustering of classified and not-classified tumors. In Figure 7c, the conversion table and Venn diagram compared the identified top 10 lncRNAs, discriminating between classified/not-classified cases and ERG/not-ERG patients. lnc-SAYSD1-1, LINC00920 and lnc-SNX10-87 were all present in the two subsets. We compared the performance of the selected features, obtaining a median accuracy of 83.5%, median sensitivity of 96.5% and 52.2% as the median specificity.

### 2.6. Co-Expression Network, Functional Enrichment and Prognostic Significance in Classified versus Nonclassified Subtypes

To gain insights into the molecular role of the selected lncRNAs, a co-expression network was built up. In Table 6, the significantly correlated lncRNA/mRNA pairs are reported, and the biological network is built up, as shown above, including genes significantly correlated to lncRNA-co-expressed mRNAs.

**Figure 8:**
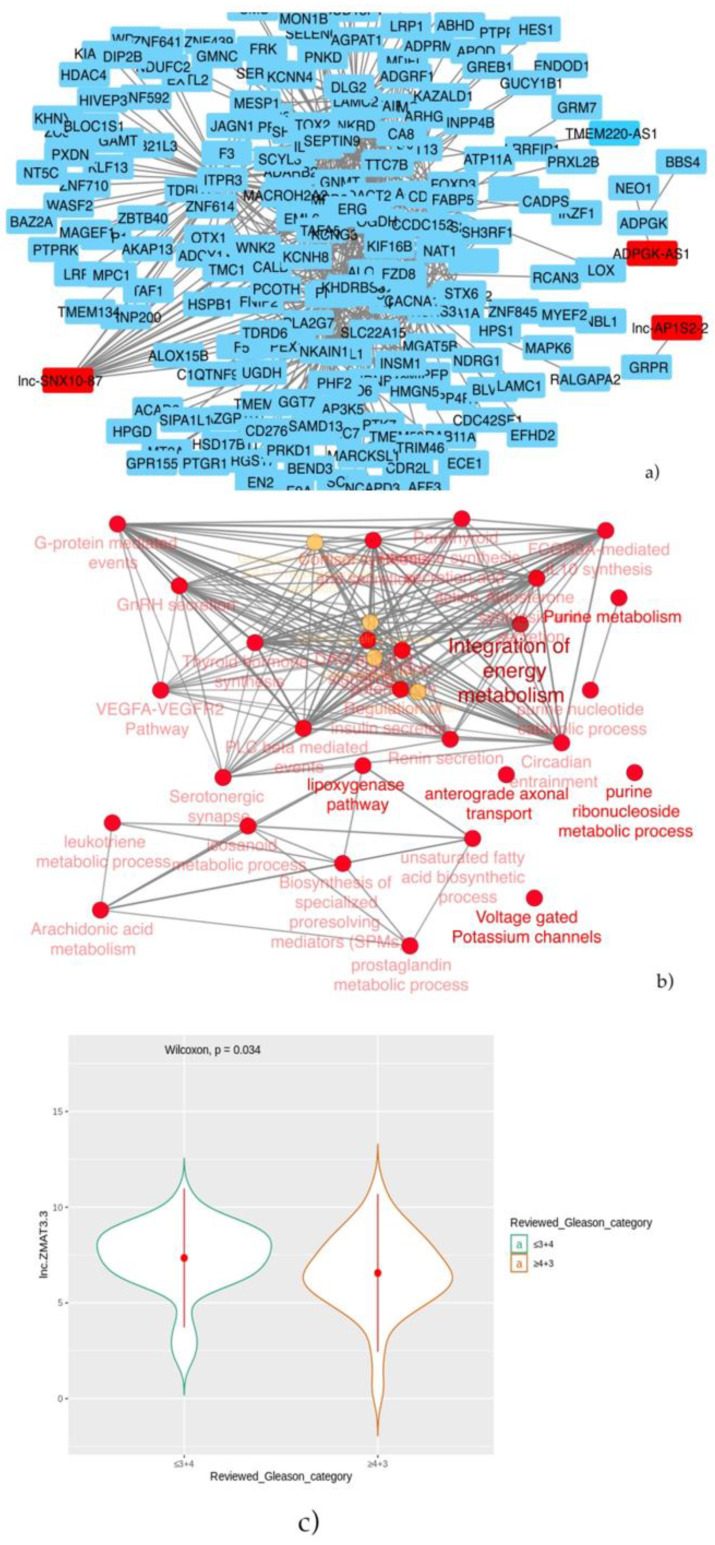
(**a**) Co-expression network, including significant correlations between lncRNAs/coding genes (red cells indicate lncRNAs with significant co-expression with genes) and (**b**) their functional enrichments. (**c**) Violin plot that shows that low-grade not-classified patients overexpress lnc-ZMAT3-3.

The lncRNAs showing significant correlations at the established cut-off were lnc-SNX10-87, ADPGK-AS1 and lnc-AP1S2-2 (Figure 8a). The functional enrichment analysis of the network evidenced the pathways involved in G-protein-mediated events, VEGFA pathways and integration of the metabolism (Figure 8b). 

Furthermore, such lncRNAs resulted inbeing not significantly correlated to PFS in patients receiving hormone therapy. However, when stratifying PCa patients according to Gleason scores, it was observed that low-grade patients (Gleason scores ≤ 3+4) have a significant higher median expression of lnc-ZMAT3-3 than high-grade patients (*p*-value = 0.034) (Figure 8c).

## 3. Discussion

In the present paper, through the application of a feature selection approach, a set of lncRNAs was identified as able to discriminate ERG-positive from other molecular subtypes and classified from not-classified. The chance to gain mechanistic insights on the biological role of the selected lncRNAs was explored through a co-expression analysis and the set-up of a biological network. Such an aspect was explored both by performing a functional enrichment of the co-expression network and the build-up of the PPI network. Moreover, the great amount of molecular data opened the chance to better stratify patients in terms of prognostic/diagnostic predictions. 

The identification of the three annotated lncRNAs LINC02418, Lnc-OR1D5-1 and Lnc-PXDN-3 discriminating the ERG-positive subtype of prostate cancer vs. others and the analysis of the co-expressed protein-coding genes and these lncRNAs revealed novel promising pharmacological targets in specific subtypes of prostate cancer. The first of them, LINC02418, we found overexpressed in the ERG-positive subtype of prostate cancer and, also, differentially expressed between colorectal cancer (CRC) tissues and noncancerous tissues [39] and upregulated in NSCLC (non-small cell lung cancer) tissues [40]. Both authors reported an active role for this lncRNA in tumorigenesis and in a CRC (colorectal cancer) model, and interestingly, Zhao demonstrated that LINC02418 upregulated maternal embryonic leucine zipper kinase (MELK) expression by acting as a ceRNA, which absorbs miR-1273g-3p [40,41]. MELK is involved in cancer cell survival and invasiveness and has been already suggested as a novel potential therapeutic target in prostate cancer [42]. Thus, in ERG-positive high-grade tumors (Gleason greater than 4+3) [42,43,44], expressing a high level of LINC02418, the MELK inhibitor OTS167 could be a promising therapeutic opportunity, because it is already utilized in clinical trials for the treatment of breast cancer and onco-hematological pathologies [45]. Another speculation, to hypothesize a novel therapeutic strategy in positive ERG prostate cancer patients, was based on the analysis of the co-expressed protein-coding genes and lncRNAs. The upregulation of the Wnt pathway and its inhibition emerged as a promising strategy against prostate cancer [46], although thinking about the most appropriate approach is imperative for the pervasive role of the Wnt pathway in normal tissue homeostasis. Several approaches have been taken into account for inhibiting the Wnt pathway, such as LGK974, a drug that targets the Wnt-specific acyltransferase porcupine, and the tankyrase inhibitor XAV939 [46], and the growing interest in the inhibition of the Wnt pathway in prostate cancer models confirms the validity of the therapeutic hypothesis. Another pathway deregulated in ERG-positive patients and correlated with the upregulation of the three lncRNAs is the purine pathway, with mainly NT5C and AMPD3 as correlated genes which opens up the possibility to utilize purine and pyrimidine antimetabolites [47] for anticancer treatments of such patients. In prostate cancer, pemetrexed, which inhibits the three enzymes used in purine and pyrimidine synthesis, has been used in combination with drugs, such as docetaxel, showing only modest clinical activity [39]. It is our opinion that a better selection of patients, stratified not only by degree of disease (Gleason value) but, also, by genomic alteration, could lead to better results in clinical trials.

Moreover, a positive prognostic role for LINC00668 and lnc-SAYSD1-1 was highlighted. In particular, we found that the overexpression of these two lncRNAs is significantly correlated with low-grade ERG-positive patients. LINC00668 is a lncRNA known for its role in cell cycle alterations. Its overexpression has already been described as a poor prognostic factor in gastric carcinoma [48], in breast cancer progression [49] and in the progression of colorectal cancer (CRC) [50]. If a role as a miR188-5p sponge is hypothesized in CRC, in gastric carcinoma, it could play a role in cyclin-dependent protein kinase inhibitors (CKIs) by means of epigenetic regulation. Both effects are contemplated in the lncRNA functions [51,52]. Our evidence on LINC00688 is in contrast with those reported in gastric, breast and colon cancers [48]. Therefore, further investigation is needed to verify the role of this lncRNA in ERG-positive prostate tumors, in which it is reported that the expression of cell cycle-related genes is negatively regulated by ERG [53]. Likewise, the lnc-SAYSD1-1 also bioinformatically allows to distinguish the ERG-positive from all the other subclasses of prostate cancer with a Gleason score ≤ 3+4. However, to date, no data have been reported on this lncRNA; therefore, we can only speculate about a possible biological role. This lncRNA maps chromosome 6 and partially overlaps the opposite strand with the DNAH8 gene (NM_001206927.2). The overexpression of this gene promotes androgen receptor activity and is associated with prostate cancer progression [54]. Therefore, we can speculate that lncSAYSD1-1 could be a cis-NAT (natural antisense transcripts) [55], and the overexpression of DNAH8 could be controlled primarily by the action of lnc-SAYSD1-1 through microRNA (miRNA) mechanisms. This would explain an overexpression of lnc-SAYSD1-1 in an early stage of the tumor, such as that represented by Gleason ≤ 3+4, while the absence in subjects suffering from cancer with Gleason ≥ 4+3 could be understood as a positive prognostic factor. 

Another interesting result from our bioinformatics analysis is that LINC00920 is overexpressed in ERG-positive patients that showed a better survival in terms of PFS to hormonal treatment, suggesting a role for this lncRNA as a predictive factor. The Lnc00920 has been reported to positively impact pathways related to the cell cycle, cell division, apoptosis and cell movement [56] by sequestering FOXO1, which functionally suppress the androgen receptor expression [57]. Then, we can hypothesize that, in low-grade ERG-positive tumors, the higher the expression of Lnc00920, the better the response to hormonal treatment, because Lnc00920 may remove the FOXO1-dependent suppression of androgen receptor expression [58].

Then, patients were stratified into two groups: prostate cancer with a known genomic alteration vs. unknown, and the co-expression analysis showed that three lncRNAs: lnc-SNX10-87, lnc-AP1S2-2 and ADPGK-AS1 have significant correlation values with other coding genes. As regards the lnc-SNX10-87 alias of LOC100506289 (uncharacterized LOC100506289), it is an RNA gene affiliated with the lncRNA class and localized on chr7:26551822-26557200 [59]. To date, nothing has been reported on the possible predictive or prognostic role of this specific lncRNA in prostate cancer. However, we found its co-expression with several protein-coding genes, among which, in particular, was the histone deacetylase 1 (HDAC1) gene, whose protein is a component of the histone deacetylase complex, a key element in the control of cell proliferation and differentiation [59]. It has been reported that HDAC activity is highly increased in metastatic cells compared with noninvasive cancer cells [60]. In particular, HDAC1 is upregulated in hormone refractory prostate cancer, and the overexpression of HDAC1 leads to an increase in prostate cancer cell proliferation [61,62]. Recently, histone deacetylase (HDAC) inhibitors have emerged as a promising new class of anticancer agents that act through a variety of mechanisms, including growth inhibition, cell cycle arrest, differentiation and apoptosis, in cancer cell lines. We suggest that these drugs may have a role in the treatment of prostate cancer patients with unknown genetic alteration. Their effectiveness has already been demonstrated in in vitro and in vivo prostate cancer studies [52,61,62]. Among these MHY219 was shown to inhibit the migration of human prostate cancer cells in the study conducted by De et al. [61]. Moreover, histone deacetylase inhibitors have been evaluated in castration-resistant prostate cancer (CRPC) or chemotherapy-resistant prostate cancer due to their effects on the expression of the androgen receptor gene. Recent clinical trials of vorinostat (ClinicalTrials.gov Identifier codes: NCT00330161 and NCT01174199), Entinostat (ClinicalTrials.gov Identifier code: NCT03829930), romidepsin and panobinostat (ClinicalTrials.gov Identifier codes: NCT00878436, NCT00493766 and NCT00663832) have provided cautious optimism towards improved outcomes using these novel therapeutic agents for CRPC patients [63]. In addition, a phase II study of SB939 in patients with recurrent or metastatic castration-resistant prostate cancer concluded in February 2020 (ClinicalTrials.gov Identifier: NCT01075308) [64]. Another lncRNA in the top 10 best-selected features by the stratification of prostate cancer patients with known genomic alteration vs. unknown is lnc-AP1S2-2. This lncRNA sequence is 454 nucleotides long and located on chrX:16153220-16165121 and has been annotated by four databases (NONCODE, LNCipedia, LncBook and GeneCards) and described by two different groups of researchers [65,66]. We found a co-expression network between lnc-AP1S2-2 and the gastrin-releasing peptide receptor (GRPR) gene, which has been extensively investigated as a molecular target in experimental anticancer therapy [67]. An important characteristic of GRPR is that it is overexpressed in prostatic tumor cells, but only low levels of receptors were found on normal prostate tissues [68,69,70,71]. Moreover, several authors studied the correlation between GRPR overexpression in PC tissues and the tumor grade or stage, leading to different results. In particular, a significant positive correlation between the GRPR expression and Gleason score in 51 PC patients in contrast to no correlation with the patient’s age, serum PSA level, pathological stage or lymph node status was demonstrated by Nagasaki et al. [72]. Beer et al. [68], who analyzed the expression of GRPR both in benign and in malignant prostate samples from 530 PC patients, found a significant inverse correlation with GRPR and a higher Gleason score, PSA value and tumor size, so GRPR was more highly overexpressed in lower-grade cancer and smaller-sized tumors. A positive association between the GRP expression and relapse or advanced tumor stages was reported by Constantinides et al. [73]. With the aim to develop a personalized management of PC patients, several ligands of GRPR, such as radiolabeled bombesin analogs (ClinicalTrials.gov Identifiers: NCT02440308 and NCT03724253) [72] able to guide the diagnosis, as well as treatment of PC, are being tested in clinical trials. However, larger prospective clinical trials are needed to strengthen the correlation between preclinical studies in mouse tumor models and the preliminary in vivo performance in cancer patients [71]. ADPGK-AS1, an antisense lncRNA gene mapped on chr15:72783884-72792963, was found differentially expressed in patients with known genomic alteration vs. unknown ones. Although nothing has been yet reported in the literature on the role of this lncRNA in the development and progression of prostate cancer, there is evidence of its role in the progression of pancreatic and gastric cancers. In particular, Song et al. [72] found that ADPGK-AS1 is involved in pancreatic cancer progression through activating zinc finger E-box-binding homeobox 1(ZEB1)-mediated epithelial–mesenchymal transition. Huang et al. [72] demonstrated that ADPGK-AS1 could promote gastric cancer progression via sponging miR-3196 and, therefore, upregulating the KDM1B gene, providing a novel prognostic biomarker and therapeutic target for GC patients. Moreover, ADPGK-AS1 was found co-expressed with the ADP-dependent glucokinase gene (ADPGK), codifying for ADPGK, which catalyzes the ADP-dependent phosphorylation of glucose to glucose-6-phosphate and may play a role in glycolysis, possibly during ischemic conditions [72].

Finally, in order to define if these lncRNAs could have a role as prognostic or predictive biomarkers, in our analysis, we found that only lnc-ZMAT3-3 is statistically higher in patients with Gleason scores ≤ 3+4 in the “not-classified” group. Thus, it could be a positive prognosticator, meaning that its levels decrease as the disease progresses. However, nothing has been reported in the literature on the role of this lncRNA in cancer. There is no other hypothesis we can formulate, due to the chromosomal position providing little food for thought and the absence of data currently present in the literature. However, this could be a stimulus for future in vitro studies. 

In conclusion, high-throughput data allowed to explore the unknown biology of tumors, increasing the burden of hypothesis-generating information. The feature selection approach used in the present study shed light on the chance to identify lncRNAs as potentially predictive biomarkers in terms of prognostication and druggable targets. Prostate cancer is a malignancy that, given its high molecular heterogeneity, could benefit from such a bioinformatic approach, targeting future studies on the selected robust lncRNAs.

## 4. Materials and Methods

### 4.1. TCGA-PRAD Data Retrieval and Preprocessing

TCGA biolinks package [74] was used to retrieve clinical data and HT-Seq count transcriptome data. Ensembl ID was converted into HUGO gene symbols by BiomaRt R package [75]. lncRNA expression data was extrapolated by the LNCipedia v5.2 conversion table [76], thus obtaining two raw read count matrices: one for mRNAs and another for lncRNAs. Molecular subtype information was retrieved by the TCGA query_subtype (tumor = “TCGA-PRAD”) function. Thus, a data frame including patient IDs and the class variable was prepared. Such a variable was ERG-positive and others (including not-ERG molecular subtypes) in the first analysis and classified/not-classified in the second analysis.

### 4.2. DaMiRseq Pipeline

#### 4.2.1. Preprocessing: Filtering by Expression, Normalization, Sample Filtering and Data Adjusting

Firstly, lncRNA expression matrix and variable information was used to create a summarized experiment (SE). The SE underwent an expression-filtering step, setting up a cut-off of at least 10 read counts in at least 70% of samples. Hyper-variantlnc RNAs were identified by calculating the coefficient of variation and excluded. The remaining features underwent normalization through a variance stabilizing transformation (“vst”). Sample–per-sample correlation was estimated, filtering out samples with a coefficient of correlation <0.9. Data adjustments were performed by the identification of surrogate variables (sv). They were identified with the “Fraction of Explained Variance” (fve) method with a cut-off of explained variance of 0.95 and used for data adjustment. The correlation between the sv and variables was verified in order to remove sv with unwanted significant correlations. As stated by Chiesa et al. [77], the correlation between sv and the “class” variable should always be not-significant.

#### 4.2.2. Feature Selection and Evaluation of Classification Performances

The aim of this step was to identify a set of robust features able to discriminate samples by the “class” label. DaMiR. F Select function of the package allowed to have an expression matrix with only informative features. Moreover, highly correlated features were also removed, setting up a correlation cut-off of 0.85. Then, the remaining features were ranked by their importance, and the first 10 were selected as the best predictors. To gain accuracy metrics, a Bootstrap resampling strategy was applied to the dataset (number of iterations = 100). The classification performances of the selected features, in terms of accuracy, sensitivity and specificity, were explored using a meta-learner—namely, “Ensemble”—that combines the RF, SVM, NB, LDA, LR and kNN classifiers.

### 4.3. Co-Expression Analysis and Network

Co-expression analysis was performed through the Hmisc R package to calculate the Pearson correlation and relative *p*-values. An expression matrix, including mRNAs and selected lncRNAs, was created and vst normalized. Then, correlations with r > |0.6| and *p*-value < 0.05 were selected. Moreover, for the network construction, genes significantly correlated to lncRNA-linked mRNAs were also included for the network build-up. Network was visualized by Cytoscape v3.8.0. The network was then functionally enriched by theClueGO app of Cytoscape, including KEGG, Gene Ontology and Reactome databases.

### 4.4. Protein–ProteinInteraction (PPI) Network and Cluster Identification

The PPI network was set up using STRING database and Cytoscape engine retrieval. Such a network was further analyzed through the MCODE [78] algorithm. Briefly, the MCODE (“Molecular Complex Detection”) algorithm is a graph-clustering algorithm able to identify highly connected regions in the PPI network. It was used as the app of Cytoscape software.

### 4.5. Statistical Analyses

PFS Kaplan-Meier curves were compared with a log-rank test by the Survival R Package. Expression values were compared through a Wilcoxon test. ggplot2 R package was used for graphs. Results were considered significant when *p*-value < 0.05.

## Figures and Tables

**Table 1:** Distribution of the molecular subtypes in the TCGA-PRAD cohort.

MolecularSubtypes	*n* (%)
ERG	151 (45.6)
ETV1	27 (8.1)
ETV4	14 (4.2)
FLI1	4 (1.2)
SPOP	38 (11.4)
FOXA1	9 (2.7)
IDH1	3 (0.9)
other	86 (25.9)

**Table 2:** Co-expressed long non-coding RNAs(lncRNAs) and coding genes.

lncRNA	Gene	r	*p*-Value
SLC5A4-AS1	CPSF1P1	0.8586	<0.0001
LINC02418	ERG	0.7761	<0.0001
lnc-PXDN-3	TPO	0.6901	<0.0001
lnc-OR1D5-1	ERG	0.6836	<0.0001
lnc-OR1D5-1	LINC02418	0.6693	<0.0001
LINC02418	CD8B2	0.6686	<0.0001
LINC02418	DACT2	0.6676	<0.0001
LINC02418	HDAC1	0.6631	<0.0001
LINC02418	ANKRD6	0.6573	<0.0001
LINC02418	OGDHL	0.6564	<0.0001
LINC02418	ALOX15	0.6530	<0.0001
LINC02418	CPNE2	0.6489	<0.0001
LINC02418	SEPTIN9	0.6437	<0.0001
LINC02418	CDH7	0.6389	<0.0001
LINC02418	ITPR3	0.6339	<0.0001
lnc-OR1D5-1	SEPTIN9	0.6311	<0.0001
LINC02418	HLA-DMB	0.6308	<0.0001
LINC02418	VSTM5	0.6233	<0.0001
LINC02418	KCNS3	0.6211	<0.0001
LINC02418	NKAIN1	0.6202	<0.0001
LINC02418	FZD8	0.6138	<0.0001
LINC02418	AMPD3	0.6120	<0.0001
lnc-PXDN-3	ERG	0.6114	<0.0001
lnc-OR1D5-1	ALOX15	0.6073	<0.0001
LINC02418	LAMC2	0.6065	<0.0001
LINC02418	SCYL3	0.6056	<0.0001
lnc-OR1D5-1	CD8B2	0.6026	<0.0001

**Table 3:** Functional enrichment of the first subcluster of the protein–protein interaction (PPI) network.

Category	Description	FDR Value
GO Function	inositol 1,4,5-trisphosphate-sensitive calcium-release channel activity	4.7 × 10^−6^
GO Function	Cation channel activity	4.7 × 10^−6^
GO Function	Ion-gated channel activity	4.7 × 10^−6^
GO Function	metal ion transmembrane transporter activity	4.7× 10^−6^
GO Function	Calcium channel activity	5.56 × 10^−6^
GO Process	Calcium ion transport	6.31 × 10^−6^
KEGG Pathways	cGMP-PKG signaling pathway	7.6 × 10^−6^
KEGG Pathways	Cellular senescence	7.6 × 10^−6^
KEGG Pathways	Vascular smooth muscle contraction	7.6 × 10^−6^
KEGG Pathways	Circadian entrainment	7.6 × 10^−6^
KEGG Pathways	Retrograde endocannabinoid signaling	7.6 × 10^−6^
KEGG Pathways	Glutamatergic synapse	7.6 × 10^−6^
KEGG Pathways	Cholinergic synapse	7.6 × 10^−6^
KEGG Pathways	Serotonergic synapse	7.6 × 10^−6^
KEGG Pathways	Dopaminergic synapse	7.6 × 10^−6^
KEGG Pathways	Insulin secretion	7.6 × 10^−6^
KEGG Pathways	GnRH signaling pathway	7.6 × 10^−6^
KEGG Pathways	Oxytocin signaling pathway	7.6 × 10^−6^
KEGG Pathways	Renin secretion	7.6 × 10^−6^
KEGG Pathways	Aldosterone synthesis and secretion	7.6 × 10^−6^
KEGG Pathways	Cortisol synthesis and secretion	7.6 × 10^−6^
KEGG Pathways	Cushing’s syndrome	7.6 × 10^−6^
KEGG Pathways	Salivary secretion	7.6 × 10^−6^
KEGG Pathways	Alzheimer’s disease	7.6 × 10^−6^
KEGG Pathways	Calcium signaling pathway	8.6 × 10^−6^
GO Function	inositol 1,4,5 trisphosphate binding	1.36 × 10^−6^
GO Process	regulation of protein secretion	1.43 × 10^−5^
Reactome Pathways	Regulation of insulin secretion	1.58 × 10^−5^
Reactome Pathways	Integration of energy metabolism	1.96 × 10^−5^
Reactome Pathways	Cardiac conduction	2.57 × 10^−5^
Reactome Pathways	CLEC7A (Dectin-1) induces NFAT activation	3.3 × 10^−5^
GO Process	Inorganic cation transmembrane transport	4.36 × 10^−5^
Reactome Pathways	Elevation of cytosolic Ca2+ levels	4.58 × 10^−5^
Reactome Pathways	Muscle contraction	4.58 × 10^−5^
Reactome Pathways	VEGFR2-mediated cell proliferation	4.58 × 10^−5^
GO Process	regulation of insulin secretion	4.82 × 10^−5^
GO Process	Calcium ion transmembrane transport	5.98 × 10^−5^
GO Process	regulation of heart contraction	6.38 × 10^−5^
Reactome Pathways	Role of phospholipids in phagocytosis	7.41 × 10^−5^
Reactome Pathways	Effects of PIP2 hydrolysis	7.62 × 10^−5^

**Table 4:** Functional enrichment of the second subcluster of the PPI network.

Category	Description	FDR Value
KEGG Pathways	Purine metabolism	9.3 × 10^−6^
Reactome Pathways	Metabolism of nucleotides	0.002
GO Process	purine nucleotide catabolic process	0.0038
GO Process	nucleoside metabolic process	0.0064
GO Process	carbohydrate derivative catabolic process	0.0064
GO Process	purine nucleotide metabolic process	0.032
GO Function	Phosphoric ester hydrolase activity	0.0323
GO Function	Hydrolase activity	0.0324

**Table 5:** Functional enrichment of the third subcluster of the PPI network.

Category	Description	FDR Value
GO Component	Wnt-Frizzled-LRP5/6 complex	7.05 × 10^−6^
ReactomePathways	RNF mutants show enhanced WNT signaling and proliferation	1.2 × 10^−5^
GO Function	Coreceptor activity involved in Wnt signaling pathway	2.89 × 10^−5^
ReactomePathways	Regulation of FZD by ubiquitination	3.37 × 10^−5^
GO Process	CanonicalWnt signaling pathway	5.31 × 10^−5^
ReactomePathways	Signaling by WNT in cancer	5.59 × 10^−5^
GO Function	Wnt-activated receptor activity	8.12 × 10^−5^
GO Function	Wnt-protein binding	1.1 × 10^−4^
GO Process	Blood vessel development	0.0013
ReactomePathways	TCF-dependent signaling in response to WNT	0.0013
GO Process	Tube morphogenesis	0.0018
KEGG Pathways	mTOR signaling pathway	0.0023
KEGG Pathways	Wnt signaling pathway	0.0023
KEGG Pathways	Breast cancer	0.0023
KEGG Pathways	Hepatocellular carcinoma	0.0023
KEGG Pathways	Gastric cancer	0.0023
ReactomePathways	Signaling by WNT	0.0023
ReactomePathways	Diseases of signal transduction	0.0029
GO Function	Signaling receptor activity	0.0032
GO Process	Non-canonical Wnt signaling pathway	0.0032
KEGG Pathways	Pathways in cancer	0.0048
GO Process	Sensory organ morphogenesis	0.0173
ReactomePathways	Disease	0.0191
GO Process	Epithelial tube morphogenesis	0.0231
GO Component	Plasma membrane part	0.0293
GO Process	Blood vessel morphogenesis	0.0328
GO Process	Regulation of protein serine/threonine kinase activity	0.0451
GO Process	Embryonic morphogenesis	0.0484

**Table 6:** Co-expressed lncRNAs and genes.

lncRNA	Gene	r	*p*-Value
lnc-AP1S2-2	GRPR	0.6863	<0.0001
lnc-SNX10-87	ALOX15	0.6841	<0.0001
lnc-SNX10-87	SEPTIN9	0.6841	<0.0001
lnc-SNX10-87	ERG	0.6795	<0.0001
lnc-SNX10-87	KCNH8	0.6795	<0.0001
lnc-SNX10-87	CDH7	0.6555	<0.0001
lnc-SNX10-87	CACNA1D	0.6539	<0.0001
lnc-SNX10-87	LAMC2	0.6372	<0.0001
lnc-SNX10-87	TTC7B	0.6349	<0.0001
lnc-SNX10-87	MCOLN3	0.6330	<0.0001
lnc-SNX10-87	KIF16B	0.6330	<0.0001
lnc-SNX10-87	FZD8	0.6286	<0.0001
lnc-SNX10-87	OGDHL	0.6286	<0.0001
lnc-SNX10-87	DACT2	0.6272	<0.0001
lnc-SNX10-87	CPNE2	0.6244	<0.0001
lnc-SNX10-87	ANKRD6	0.6234	<0.0001
lnc-SNX10-87	EML6	0.6161	<0.0001
lnc-SNX10-87	ABCC8	0.6059	<0.0001
lnc-SNX10-87	NKAIN1	0.6059	<0.0001
ADPGK-AS1	ADPGK	−0.6047	<0.0001
lnc-SNX10-87	HDAC1	−0.6154	<0.0001
lnc-SNX10-87	KCNS3	−0.6166	<0.0001
lnc-SNX10-87	CD8B2	−0.6204	<0.0001
lnc-SNX10-87	HLA-DMB	−0.6444	<0.0001
lnc-SNX10-87	ITPR3	−0.6802	<0.0001

## Data Availability

Data are available in GDC data portal (https://portal.gdc.cancer.gov/) (accessed on 23 February 2021).

## Abbreviations

PCa	Prostate cancer
PSA	Prostate Specific Antigen
lncRNA	Long non-coding RNA
CRPC	Castration resistant prostate cancer
ADT	Deprivation Androgenic
MDS plot	Multidimensional scaling plot
RF	Random Forest
SVM	Support Vector Machine
NB	Naïve Bayes classifiers
LDA	Latent Dirichlet allocation
LR	Linear Regression
kNN	k-nearest neighbors algorithm
PPI	protein–protein interaction
PFS	Progression-Free Survival
CRC	colorectal cancer
CKIs	protein kinase inhibitors
vst	Variance stabilizing transformation
sv	Surrogate variables
fve	Fraction of Explained Variance
